# Microbiota–Gut–Brain Axis Disruption, Neuroinflammation, and Potential Antioxidant-Based Treatments in Metabolic Diseases

**DOI:** 10.3390/antiox15040522

**Published:** 2026-04-21

**Authors:** Jazmín Carro-Rodríguez, Gabriela Ibáñez-Cervantes, Noemí Cárdenas-Rodríguez, Iván Ignacio-Mejía, Exsal Manuel Albores-Méndez, Blanca Rosalba Pardo-Pacheco, Verónica Fernández-Sánchez, Ana María Balboa-Verduzco, Cecilia Adame, Eleazar Lara-Padilla, Javier Mancilla-Ramírez, Roberto Medina-Santillán, Macarena Montoya-Olvera, Alfredo Leonardo Cortes-Algara, Saúl Gómez-Manzo, Beatriz Hernández-Ochoa, Heliodoro Moya-Amaya, Cindy Bandala

**Affiliations:** 1 Laboratorio de Neurociencia Traslacional, Escuela Superior de Medicina, Instituto Politécnico Nacional, Mexico City 11340, Mexico; jcarror2400@alumno.ipn.mx (J.C.-R.); ivanignacio402@gmail.com (I.I.-M.); mad2300@alumno.ipn.mx (C.A.); 2 Laboratorio de Microbiología y Biología Molecular, Departamento de Desarrollo de Tecnologías, Centro Interdisciplinario de Ciencias Marinas, Instituto Politécnico Nacional, La Paz 23096, Baja California Sur, Mexico; gibanezc@ipn.mx; 3 Laboratorio de Neurociencias, Instituto Nacional de Pediatría, Secretaría de Salud, Mexico City 04530, Mexico; noemicr2001@yahoo.com.mx; 4 Sección de Investigación, Escuela Militar de Graduados de Sanidad, Centro de Investigación y Desarrollo del Ejército y Fuerza Aérea Mexicanos, Universidad del Ejército y Fuerza Aérea, Mexico City 11200, Mexico; exsalalbores@gmail.com; 5 Nutriología Clínica, Servicio de Endocrinología y Bariatria, Hospital Juárez de México, Mexico City 07760, Mexico; blaropp72@yahoo.com.mx; 6 Facultad de Estudios Superiores de Iztacala, Universidad Nacional Autónoma de México, Mexico City 54090, Mexico; veronica.fernandez@salud.gob.mx; 7 Sección de Estudios de Posgrado e Investigación, Escuela Superior de Medicina, Instituto Politécnico Nacional, Mexico City 11340, Mexico; abalboav@ipn.mx (A.M.B.-V.); elarap@ipn.mx (E.L.-P.); jmancillar@ipn.mx (J.M.-R.); robertomedsan@yahoo.com (R.M.-S.); 8 Secretaría de Salud del Estado de México, Dirección del Instituto de Salud del Estado de México, Toluca 50070, State of Mexico, Mexico; macarena.montoya@edomex.gob.mx; 9 Dirección General del Instituto Materno Infantil del Estado de México, Toluca 50170, State of Mexico, Mexico; drcortesimiem@gmail.com; 10 Laboratorio de Bioquímica Genética, Instituto Nacional de Pediatría, Secretaría de Salud, Mexico City 04530, Mexico; saulmanzo@ciencias.unam.mx; 11 Laboratorio de Investigación en Ciencias Ómicas y Epidemiología Microbiana, Hospital Infantil de México Federico Gómez, Secretaría de Salud, Mexico City 06720, Mexico; beatrizhb_16@ciencias.unam.mx; 12 Grupo de Investigación CTS-595, Centro de Investigación en Rendimiento Físico y Deportivo (CIRFD), Universidad Pablo de Olavide, 41089 Sevilla, Spain; helioma91@gmail.com

**Keywords:** microbiota–gut–brain axis, neuroinflammation, metabolic diseases, oxidative stress, antioxidants

## Abstract

Metabolic diseases are strongly associated with chronic systemic inflammation and oxidative stress, which disrupt the microbiota–gut–brain (MGB) axis and promote neuroinflammation. Dysbiosis favors the release of proinflammatory metabolites, reactive oxygen species (ROS), and lipopolysaccharides (LPS), increasing intestinal permeability and triggering systemic immune responses that reach the central nervous system (CNS) through a weakened blood–brain barrier (BBB). This review summarizes current knowledge on the pathophysiological mechanisms linking the MGB axis, metabolic disorders, and neuroinflammation, as well as the therapeutic potential of antioxidants. A literature search was conducted in PubMed, Web of Science, Scopus, and ScienceDirect and included original research articles, reviews, clinical trials, and meta-analyses related to microbiota, neuroinflammation, oxidative stress, and antioxidant interventions. Evidence indicates that dysbiosis exacerbates metabolic dysfunction by activating the nuclear factor kappa B (NF-κB) and NOD-like receptor family pyrin domain containing 3 (NLRP3) inflammasome pathways, while excessive ROS production impairs mitochondrial function, neuronal survival, and cognitive processes. Antioxidant strategies, including polyphenols, omega-3 fatty acids, curcumin, vitamins C and E, and probiotics, can restore microbial diversity, reinforce intestinal and BBB integrity, and modulate oxidative and inflammatory signaling. In conclusion, supplements and bacteria with antioxidant properties show promising therapeutic effects by targeting oxidative stress mechanisms involved in metabolic diseases and their pathological consequences, such as dysbiosis and neuroinflammation.

## 1. Introduction

Metabolic diseases are complex disorders that have shown an increasing global prevalence. Despite available therapeutic strategies, they remain a major global public health problem [1]. In this review, we include obesity, T2DM, dyslipidemia, and metabolic syndrome (MS), as these are closely related conditions that share diverse pathophysiological mechanisms, including oxidative stress, low-grade systemic inflammation, insulin resistance, and endothelial dysfunction, among others. These mechanisms also contribute to the modulation of intestinal dysbiosis, BBB integrity, and neuroinflammation [2,3,4,5,6,7,8,9]. In this context, metabolic diseases can trigger dysbiosis and neuroinflammation in a reinforcing circuit between them [8,10,11].

The MGB axis is a bidirectional communication system that connects the gastrointestinal tract, the gut microbiota, and the CNS. Several studies have shown that the MGB axis can regulate appetite and satiety [12,13,14] as well as processes related to cognition, attention and memory [15,16,17], which are also associated with neuroinflammation [18,19,20,21]. Neuroinflammation is considered a comorbidity of obesity and other metabolic diseases and may arise through various pathophysiological mechanisms, including proinflammatory adipokines, insulin resistance, excess lipids and, importantly, dysbiosis [10,22,23]. However, oxidative stress represents a key pathophysiological mechanism. Redox imbalance is an important therapeutic target, as it is both a cause and a consequence of metabolic dysfunction, dysbiosis, and neuroinflammation. In this regard, antioxidants have been proposed as complementary therapeutic tools due to their ability to modulate neuroinflammation [24,25], restore biological barriers [26], and promote a more stable microbiota [27]. The potential antioxidants (both bioactive molecules and probiotics), whether used alone or in combination, that may positively influence the treatment of metabolic diseases and their comorbidities, such as dysbiosis and neuroinflammation, remain to be fully elucidated.

In this review, we analyze the role of oxidative stress in metabolic diseases related to neuroinflammation and intestinal dysbiosis, as well as possible antioxidant strategies for their treatment.

## 2. Methodology

This narrative review focuses on two complementary aspects: (1) the pathophysiological mechanisms linking oxidative stress, gut dysbiosis, and neuroinflammation in metabolic diseases; and (2) antioxidant- and microbiota-targeted therapeutic strategies with potential clinical relevance. For the selection and analysis of the literature, we consulted the following sources: PubMed, Web of Science, Scopus, ScienceDirect, SciFinder, ProQuest, EBSCO, Google Scholar, and ClinicalTrials.gov. Supporting resources for compound/pathway information and toxicology background included PubChem, NCBI Bookshelf, DrugBank, and LiverTox.

We considered original research articles, reviews, systematic reviews, meta-analyses, clinical trials, and book chapters. Searches were conducted using individual and combined keywords such as “gut microbiota,” “microbiota-gut-brain axis,” “neuroinflammation,” “metabolic disease,” “obesity,” “type 2 diabetes mellitus,” “dyslipidemia,” “metabolic syndrome,” “antioxidants,” “oxidative stress,” “inflammatory markers,” “prebiotics,” and “probiotics”. After selecting studies relevant to both pathophysiological mechanisms and antioxidant-based interventions, 212 references were selected for in-depth analysis. The searches were conducted from 1980 to 2026. Relevant studies on intestinal dysbiosis, oxidative stress, neuroinflammation, and metabolic diseases were included, whereas irrelevant reports, duplicate records, and studies lacking clear mechanistic or clinical relevance were excluded.

## 3. Pathophysiological Mechanisms Related to Neuroinflammation and Dysbiosis in Metabolic Diseases

Metabolic diseases have been linked to dysregulation of appetite and satiety, as well as cognitive and memory impairment, increased aggression, anxiety, depression, and social isolation [28,29]. Neuroinflammation, or gliosis, has been identified as an underlying factor contributing to these pathological conditions [30]. The pathophysiological mechanisms have been associated with low-grade chronic inflammation, redox imbalance, and dysbiosis [31,32]. It is important to recognize that metabolic disease, neuroinflammation, and dysbiosis are interconnected pathological conditions that interact with one another, with oxidative stress and inflammation acting as their primary axes [18,32,33].

### 3.1. Neuroinflammation in Obesity, T2DM, Dyslipidemia, and Metabolic Syndrome

Neuroinflammation in metabolic diseases is a multifactorial process involving a complex network of molecular signals, including impaired neuroprotective mechanisms and alterations in CNS homeostasis and BBB integrity [8]. The importance of the BBB lies in the fact that it is a dynamic structure composed primarily of endothelial cells connected by tight junctions, pericytes, astrocytes, and other components of the neurovascular unit [34]. Its function is to selectively regulate the passage of molecules, cells, and signals from the circulation into the CNS, thereby maintaining cerebral homeostasis; protecting nervous tissue from toxins, pathogens, and peripheral inflammatory mediators; and preserving proper neuronal function [34,35]. BBB destabilization is promoted by endothelial oxidative stress [36], altered expression of tight junction proteins [37], inflammatory activation of the neurovascular unit [38], and increased permeability to circulating cytokines, lipids, and endotoxins [8,31]. Once this barrier is compromised, peripheral inflammatory mediators gain greater access to the brain parenchyma, facilitating gliosis and sustained neuroinflammation [8,39], which involves the persistent activation of microglia and astrocytes, with harmful effects in the medium and long term [40].

Another mechanism that contributes to neuroinflammation is gut dysbiosis, which acts as a trigger by promoting NF-κB and NLRP3 inflammasome activation. This leads to the systemic release of cytokines such as tumor necrosis factor alpha (TNF-α), interleukin-1 beta (IL-1β), and interleukin-6 (IL-6), as well as chemokines that weaken the BBB [41,42]. In addition to this immune response, communication within the MGB axis is altered by changes in chemical mediators [7] and cholesterol metabolites [43]. These mediators and proinflammatory signals can cause significant shifts in extracellular ion levels, especially by changing potassium and calcium gradients—a key process involved in activating and directing microglia toward a proinflammatory state [44,45]. Therefore, disruption of ionic and chemical balance, intensified by vagus nerve signaling [46,47], sustains chronic gliosis and neurological damage. Simultaneously, repression of essential pathways is observed, manifested as decreased neuronal plasticity [48].

Obesity is a chronic metabolic disease characterized not only by excessive accumulation of adipose tissue but also by sustained activation of systemic and central inflammatory responses [31,49,50]. Visceral adipose tissue functions as an active endocrine and immunological organ that secretes proinflammatory adipokines, including leptin, TNF-α, and IL-6, which are related to neuroinflammation [51]. These adipokines are secreted by hypertrophic adipocytes, primarily from visceral fat, and are transported through the systemic circulation, preferentially affecting brain regions with high vascular exposure and specialized characteristics of the BBB [52]. This vascular configuration, along with relatively high blood flow, a more permeable BBB (secondary to endothelial inflammation, oxidative stress, and alterations in tight junction proteins), and its high exposure to peripheral endocrine and nutritional signals, explains why adipokines from visceral adipose tissue more readily access hypothalamic neurons [41,51]. Furthermore, due to their distribution throughout the systemic circulation, these mediators also reach regions of the hedonic brain, such as the ventral tegmental area and the nucleus accumbens which, although exhibiting a tighter BBB, can be influenced by active transport mechanisms and by increased permeability induced by inflammation [53]. At a functional level, leptin modulates the electrical activity and synaptic plasticity of proopiomelanocortin (POMC) neurons and neuropeptide Y/Agouti-related protein (NPY/AgRP) neurons in the hypothalamus, in addition to influencing dopaminergic signaling in reward circuits [54,55,56,57]. Elevated leptin levels in obese individuals also potentiate this activation via Janus kinase 2/signal transducer and activator of transcription 3 (JAK2/STAT3), perpetuating the proinflammatory state and interfering with satiety signals [58]. In parallel, cytokines such as IL-6 and TNF-α induce microglial activation and neuroinflammation, reach the hypothalamus and reprogram microglia toward an M1 phenotype, characterized by the sustained release of IL-1β and interleukin-18 (IL-18), thereby impairing leptin and insulin signaling [59]. Taken together, the combination of high vascular accessibility and the expression of specific receptors allow circulating factors derived from visceral adipose tissue to simultaneously alter homeostatic and hedonic circuits, favoring appetite dysregulation, loss of satiety signaling, and increased food craving or reward-driven food consumption.

Another mechanism linked to neuroinflammation in obesity is a high-fat diet (HFD) [60]. Studies in animal models have shown that HFD induces microglial activation and astrogliosis in the arcuate nucleus of the hypothalamus, even before obesity becomes clinically apparent [61]. Although HFD models do not fully replicate the endocrine complexity of visceral adipose tissue, they provide complementary mechanistic evidence linking metabolic inflammation to BBB dysfunction and neural damage. HFD consumption increases ROS and enhances the production of adipokines such as TNF-α, IL-6, and IL-1β, which maintain low-grade systemic inflammation and a redox imbalance [62,63]. At the same time, exposure to an HFD has been associated with altered expression of BBB-related proteins, including tight junction proteins such as occludin, reduced barrier integrity in hypothalamic regions, and increased permeability to low- and high-molecular-weight markers, indicating early neurovascular dysfunction [64,65]. Experimental evidence also shows that animals fed an HFD develop adiposity, hyperleptinemia, insulin resistance, oxidative stress, and endotoxemia-related inflammation [65,66], factors that may contribute to endothelial dysfunction in the BBB [67,68,69,70]. Chronic activation of glial and astroglia, also observed in humans using neuroimaging techniques, has been associated with brain insulin resistance and deterioration of neural circuits involved in appetite regulation [50,61].

Finally, a decrease in the levels of neurotrophic factors such as BDNF has also been observed as part of neuroinflammation in obesity, which compromises neuroplasticity and synaptic function, contributing to cognitive and emotional alterations in individuals with obesity [48]. BDNF-mediated changes occur primarily in the hypothalamus (e.g., the arcuate nucleus), as well as in reward-related regions such as the hippocampus, ventral tegmental area, and nucleus accumbens [71,72]. These changes include both structural plasticity (dendritic remodeling and changes in synaptic density) and functional plasticity (modifications in neuronal activity, neurotransmitter signaling, and circuit dynamics) [73].

In T2DM, chronic hyperglycemia and insulin resistance are the main factors linked to neuroinflammation [74]. Hyperglycemia, through pathways such as the polyol pathway, advanced glycation end products (AGEs), and protein kinase C (PKC) activation, increases the activity of the NF-κB pathway, thereby enhancing the production of IL-1β, TNF-α and ROS while simultaneously reducing the activity of antioxidant enzymes such as superoxide dismutase (SOD) and catalase (CAT), ultimately leading to neuronal and glial damage [75]. Under normal conditions, insulin exerts neuroprotective and anti-inflammatory effects in the CNS. In insulin resistance, these effects are diminished, limiting neuronal survival and promoting glial activation and chronic inflammation [67,76,77,78]. Microglia and astrocytes are the primary cellular elements responsible for the release of inflammatory mediators in response to insulin resistance [67,74]. These glial cells exhibit insulin resistance mechanisms analogous to those observed in neurons, where impaired insulin receptor signaling disinhibits the activation of proinflammatory pathways such as NF-κB and c-Jun N-terminal kinase (JNK) [9,75]. The gastrointestinal microbiota directly influences this central insulin resistance through the release of LPS, which activates Toll-like receptor 4 (TLR4) in glial cells, exacerbating the production of cytokines (TNF-α, IL-6) that interfere with neuronal insulin signaling [6,79]. This process promotes degenerative neuronal changes, such as reduced BDNF levels and loss of synaptic integrity, linking dysbiosis to the cognitive impairment observed in diabetic patients [48,78]. Furthermore, central insulin resistance impairs the normal anti-inflammatory effects of this hormone, exacerbating gliosis and compromising brain structural integrity [78]. The consequences of neuroinflammation in diabetes include complications such as diabetic neuropathy, a disorder that affects peripheral and central nerves, causing pain, numbness, and loss of sensation [80]. In addition, diabetes increases the risk of cognitive impairment and dementia, including Alzheimer’s disease, due to chronic inflammation and synaptic dysfunction in the brain [81,82].

Dyslipidemia, characterized by elevated levels of total cholesterol, low-density lipoproteins (LDL), and triglycerides, together with reduced high-density lipoproteins (HDL), is associated with a systemic inflammatory state that includes neuroinflammation [83]. Excess free fatty acids induce the production of proinflammatory cytokines that can cross the BBB and contribute to neuroinflammation [49,84]. This inflammatory milieu is promoted by the accumulation of altered lipid species, particularly oxidized LDL (oxLDL) and triglyceride-rich lipoproteins, which activate endothelial cells, macrophages, and other immune effectors, increasing the production of mediators such as TNF-α, IL-6, IL-1β, monocyte chemoattractant protein-1 (MCP-1), and ROS [85,86]. Through endothelial dysfunction and BBB disruption, these lipotoxic and inflammatory signals facilitate the entry of peripheral mediators into the brain, promoting microglial and astrocytic activation and sustaining chronic neuroinflammation [5,87,88]. In addition, altered cholesterol metabolism in the CNS can promote the accumulation of toxic metabolites such as 24S-hydroxycholesterol, which intensifies oxidative stress and neuronal inflammation [43]. Excess free fatty acids induce the production of proinflammatory cytokines that can cross the BBB and contribute to neuroinflammation [49,84]. This inflammatory environment compromises synaptic plasticity and neuronal homeostasis, contributing to cognitive impairment and an increased risk of neurodegenerative conditions such as Alzheimer’s disease [89].

MS, a cluster of disorders that includes abdominal obesity, insulin resistance, dyslipidemia, and hypertension, increases the risk of cardiovascular disease and T2DM [90]. As expected, MS has also been linked to neuroinflammation [2,8,9,42,75,91], affecting brain energy homeostasis, including regulatory centers of the hypothalamus and the hedonic brain, as we mention previously [41,92]. Additionally, hypertension, as a central component of MS, contributes to the development of neuroinflammation through multiple hemodynamic and immunometabolic mechanisms, such as endothelial dysfunction and increased ROS production, which favors increased BBB permeability [93]. This process is further potentiated by the activation of the renin–angiotensin system, particularly through angiotensin II, which stimulates ROS production and activates proinflammatory pathways such as NF-κB in endothelial cells and microglia [94]. Consequently, microglial activation and astrogliosis occur, accompanied by the release of inflammatory mediators (IL-1β, TNF-α, and IL-6) that alter neuronal function and synaptic homeostasis, as previously described. In the context of MS, these effects are amplified by the coexistence of insulin resistance, visceral adiposity, and a low-grade systemic inflammatory state, which perpetuates neurovascular dysfunction and promotes neurodegeneration and cognitive decline [30,74,76].

### 3.2. Dysbiosis in Metabolic Disease

The MGB axis participates in the regulation of physiological processes related to metabolism, inflammation, and the response to oxidative stress (redox imbalance) [95,96]. It is important to note that oxidative stress is not only a consequence but also a key pathophysiological nexus linking metabolic inflammation with dysfunction of the MGB axis [33]. Alteration of the MGB axis is a key factor in the pathophysiology of metabolic diseases [97,98] and is associated with dysbiosis, which is characterized by reduced microbial diversity and an increased proportion of pathogenic bacteria [32]. Changes in the microbiota lead to the production of proinflammatory metabolites, such as LPS, which can cross the damaged intestinal barrier and activate the immune system. Once in the bloodstream, they can cross the BBB and activate microglia, triggering neuroinflammation [99]. Under homeostatic conditions, the microbiota produces metabolites such as short-chain fatty acids (SCFAs), which regulate neuroinflammation and oxidative stress [100,101]. However, in dysbiosis, the microbiota reduces SCFA production and increases the generation of proinflammatory metabolites and ROS, contributing to the development and progression of metabolic diseases such as obesity [97]. SCFAs, including acetate, propionate, and butyrate, act as ligands for G protein-coupled receptors (GPCRs), such as GPR41 and GPR43 [102]. Specifically, GPR41/43 is involved in energy regulation in response to SCFAs [101], particularly in enteroendocrine cells, where it promotes the secretion of satiety signals and adjusts the response of the hypothalamic–pituitary–adrenal axis [46]. In contrast, GPR43 modulates the activity of NF-κB through β-arrestin 2 (βarr2), reducing the production of proinflammatory cytokines [102] and improving intestinal barrier integrity [103].

Obesity has been described as inducing intestinal dysbiosis, characterized by reduced microbial diversity and altered proportions of major bacterial phyla. These alterations include increased *Firmicutes*, members of the class *Negativicutes*, and the genus *Lachnoclostridium*, along with decreased levels of *Bacteroides thetaiotaomicron* [104,105], *Akkermansia muciniphila* [106,107], *Faecalibacterium prausnitzii* and *Roseburia* spp., which have been described as the main strains responsible for butyrate production [108]. The reduction or excess of certain microbial strains modifies the production of SCFAs (mainly acetate, propionate, butyrate), which are sensed by enteroendocrine cells and vagus nerve cells, thereby influencing the hypothalamus [109]. At the same time, the increased intestinal permeability characteristic of obesity facilitates the passage of LPS into the circulation, triggering low-grade systemic inflammation that aggravates insulin resistance and activates cerebral microglia, promoting neuroinflammatory processes [110]. In addition, the microbiota influences the endocrine axis by modulating hormones such as ghrelin, leptin, peptide YY, and glucagon-like peptide-1 (GLP-1) [111].

In models of obesity and T2DM, intestinal dysbiosis promotes the growth of Gram-negative bacteria, which release LPS. These molecules are associated with increased levels of inflammatory biomarkers through activation of the immune system via Toll-like receptor 4/myeloid differentiation factor 2 (TLR4-MD-2), thereby triggering a systemic inflammatory response [79,112]. Intestinal dysbiosis and increased LPS production by Gram-negative bacteria, such as *Escherichia coli*, *Enterobacter massiliensis*, *Salmonella enterica*, and *Shigella flexneri*, activate the NLRP3 inflammasome, leading to increased production of IL-1β and IL-18 [98,113,114,115]. On the other hand, activation of TLR4 by LPS induces receptor dimerization, allowing Toll-like receptor-associated adaptor protein (TIRAP) and myeloid differentiation primary response protein 88 (MyD88) to activate interleukin receptor-associated protein kinases (IRAK1/2) and the TNF receptor-associated factor 6 (TRAF6) complex. This signaling cascade activates the NF-κB pathway and increases TNF-α and interleukin-6 (IL-6) levels [110]. Likewise, the vagus nerve is highly relevant in inflammation associated with the MGB axis, as it transmits inflammatory signals from the intestine to the brain, where it promotes microglial activation [47]. This activation contributes to neuroinflammation but is also associated with alterations in appetite control and energy homeostasis [116]. Intestinal dysbiosis in T2DM is associated with a decrease in butyrate-producing bacteria, including reduced levels of *Bacteroides fragilis* and other key species, and this deficit affects ROS production. In diabetic dysbiosis, low butyrate availability alleviates the inhibition of histone deacetylase 3 (HDAC3), leading to increased expression of NADPH oxidase 4 (NOX4), an enzyme responsible for ROS generation. This increase in ROS is associated with an altered inflammatory state and changes in colon permeability and function [117]. In addition, increased ROS production and other markers of oxidative stress have been positively correlated with a greater abundance of *Escherichia coli* and *enterococci* and negatively correlated with the presence of *lactobacilli* [118]. Elevated ROS levels in the intestine and adipose tissue, together with alterations in oxidative stress biomarkers such as thiobarbituric acid reactive substances (TBARS) and antioxidant enzymes including SOD, CAT, and glutathione peroxidase (GPx), are associated with mitochondrial dysfunction. This alteration compromises adenosine triphosphate (ATP) production, promotes the release of proinflammatory cytokines, and can lead to cellular damage and apoptosis [119,120].

**Figure 1:**
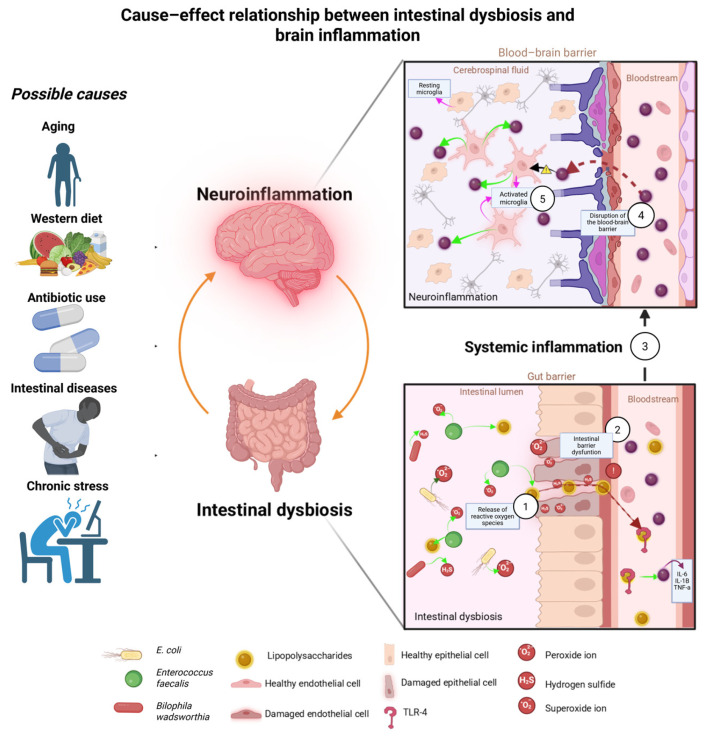
Cause–effect relationship between intestinal dysbiosis and brain inflammation: The left panel illustrates potential triggers of dysbiosis, such as aging, a Western diet, antibiotic use, intestinal diseases, and chronic stress. Below, dysbiosis promotes the release of ROS (O_2_^●—^, H_2_S) and LPS by bacteria (1), and this new environment degrades the intestinal barrier (2), allowing these molecules to pass through the damaged mucosa into the bloodstream, where they bind to receptors such as TLR4 and trigger systemic inflammation (3). Subsequently, these molecules enter through a compromised BBB (4), activating microglia, which releases even more inflammatory molecules (5), perpetuating neuroinflammation. Created in BioRender. Bandala, C. (2026) https://BioRender.com/7x6m0mz (accessed on 1 March 2026).

The gut microbiota modulates the expression of antioxidant enzymes. Under conditions of dysbiosis, these antioxidant enzymes are compromised, increasing susceptibility to oxidative damage [119,121]. ROS, such as superoxide anion (O_2_^●—^) and hydrogen peroxide (H_2_O_2_), activate proinflammatory pathways, including NF-κB, which regulates the expression of genes involved in cell survival [122], and NLRP3, which is related to the pathogenesis of inflammatory diseases [123]. In turn, inflammatory cytokines such as TNF-α and IL-6 further increase ROS production [121,124]. This cycle perpetuates metabolic dysfunction. Oxidative stress induces deoxyribonucleic acid (DNA) damage and promotes cellular senescence in metabolically active tissues such as the liver and adipose tissue [125]. This contributes to insulin resistance and hepatic steatosis [126,127], and persistent oxidative stress has also been linked to neurodegeneration [128]. Figure 1 illustrates the causal relationship between intestinal dysbiosis and neuroinflammation.

## 4. Antioxidant Strategies Targeting Gut Dysbiosis and Neuroinflammation in Metabolic Diseases

### 4.1. Redox-Modulating Bacteria: Pro-Oxidant vs. Antioxidant Profiles

Oxidative stress within the host is closely linked to the composition of the gut microbiome [129]. On the one hand, certain Gram-negative and sulfate-reducing bacteria produce endotoxins or hydrogen sulfide (H_2_S), which activate inflammatory pathways and damage the mucosal barrier, contributing to intestinal dysfunction and systemic inflammation [130]. In contrast, other microorganisms produce antioxidant enzymes such as SOD, generate butyrate, or synthesize reduced glutathione (GSH), processes that neutralize free radicals and strengthen mucosal integrity [131]. This microbial duality between pro-oxidants and antioxidants reveals potential therapeutic targets for restoring redox balance. Table 1 details some characteristics of pro-oxidant bacteria.

Due to the presence of pro-oxidant bacteria that form part of the gut microbiome and can induce systemic inflammatory conditions, including processes in the CNS such as neuroinflammation [138], recommending probiotics with antioxidant properties, which include free radical scavengers and antioxidant enzymes, may be a therapeutic resource for these pathological conditions [24]. Table 2 shows the antioxidant bacteria that have demonstrated therapeutic effects in models of dysbiosis, inflammatory conditions, and metabolic disorders.

Despite the limited information available on gut microbiota bacteria with antioxidant capabilities, evidence shows that they have therapeutic potential in different clinical contexts; for example, in metabolic diseases and their impact on neuroinflammation and dysbiosis.

### 4.2. Antioxidant Supplementation as a Therapeutic Strategy

Antioxidant supplements have been shown to have therapeutic implications for metabolic conditions such as obesity, neuroinflammation, and gut dysbiosis [10,25,27,148]. Polyphenols present in foods such as green tea, berries, and cocoa reach the colon, where they are metabolized by the gut microbiota into low-molecular-weight phenolic compounds with high bioavailability and potent antioxidant activity [149]. These compounds modulate bacterial composition, favoring the growth of genera such as *Lactobacillus*, *Bifidobacterium*, and *Akkermansia*, which in turn strengthen the intestinal barrier and promote the production of SCFAs (butyrate and propionate) with local anti-inflammatory and antioxidant effects [149,150]. Structurally, the –OH groups of polyphenols capture free radicals and can activate the nuclear factor erythroid 2-related factor 2 (Nrf2) pathway in epithelial cells, increasing the expression of enzymes such as SOD, CAT, and GPx [151]. Resveratrol and curcumin are examples of polyphenols that inhibit proinflammatory pathways such as NF-κB, thereby reducing the production of inflammatory cytokines, including TNF-α and IL-1β [25]. This effect not only reduces neuroinflammation but also improves insulin sensitivity and metabolic function in models of obesity and metabolic diseases [36].

Polyphenols, vitamin C, vitamin E, and GSH act directly on ROS, reducing their concentration and preventing or mitigating oxidative damage to neurons and glial cells. In particular, vitamin E, a fat-soluble antioxidant, protects cell membranes from lipid peroxidation, a process common in diabetes and dyslipidemia [152].

**Figure 2:**
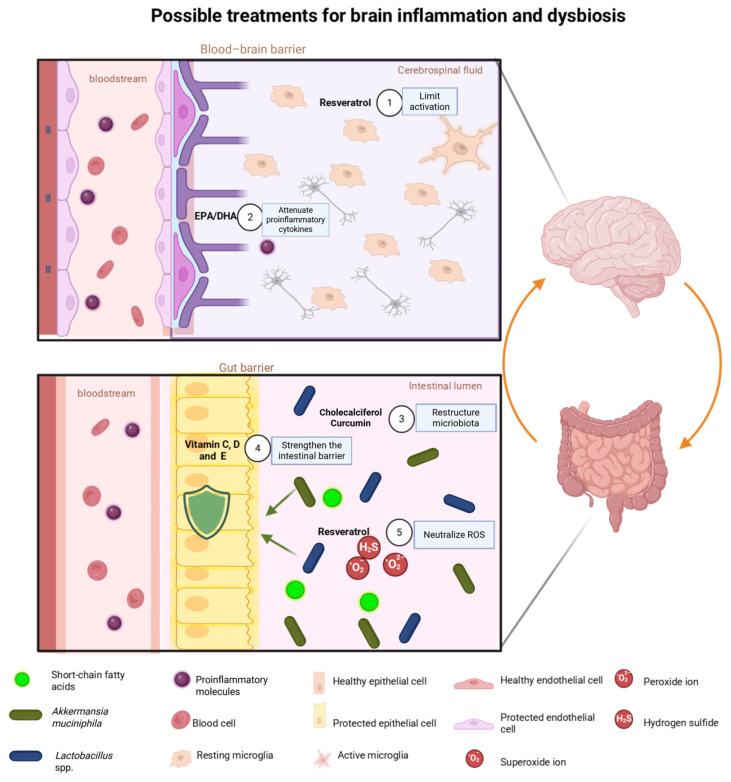
Proposed antioxidant-based strategies targeting gut dysbiosis and neuroinflammation in metabolic diseases. The diagram summarizes representative compounds and their mechanisms of action: Numbers 1–5 in the figure indicate the corresponding mechanisms described below. 1. Resveratrol activates Nrf2 to increase SOD, catalase, and GPx, limiting microglial activation in the CNS. 2. EPA/DHA are incorporated into the cell membrane, displacing proinflammatory precursors and generating resolvins that reduce the release of TNF-α and IL-1β in the BBB. 3. Cholecalciferol and curcumin reorganize the microbiota in favor of *Lactobacillus* and *Akkermansia*, inhibit NF-κB signaling, and suppress TNF-α and IL-6, thereby strengthening the intestinal barrier. 4. Vitamins C, D, and E scavenge free radicals, mutually regenerate their active form, and interrupt lipid peroxidation while promoting SCFA production. 5. Resveratrol neutralizes ROS (·O_2_^−^, H_2_O_2_) in the intestinal lumen by donating electrons to free radicals and activating Nrf2 to increase antioxidant enzymes. Overall, these strategies provide simultaneous protection of the intestinal mucosa and the BBB, reducing proinflammatory molecules and reinforcing epithelial and endothelial integrity. Created in BioRender. Bandala, C. (2026) https://BioRender.com/kvl0gqx (accessed on 1 March 2026).

Table 3 and Figure 2 summarize antioxidant compounds with effects on the microbiota and their impact on neuroinflammation.

### 4.3. Combined Antioxidant and Probiotic Strategies

The therapeutic strategy of combining probiotics with antioxidants may be promising for managing dysbiosis and neuroinflammation in metabolic diseases; however, much of the available evidence derives from related clinical contexts (e.g., psychiatric, cardiovascular, or endocrine conditions) and should therefore be interpreted as indirect but mechanistically relevant support. Although conclusive evidence remains limited, studies conducted in both experimental models and humans suggest that this approach may exert beneficial effects. In experimental models, *Lactobacillus paracasei* L14 supplementation (10^10^ colony-forming units, CFU) has been shown to reduce lipid peroxidation and increase the activity of antioxidant enzymes, including CAT, GPx and SOD, thereby attenuating inflammation and intestinal dysbiosis. These findings suggest systemic regulatory effects in a streptozotocin (STZ)-induced diabetes model in rats [167,168,169]. In a STZ plus nicotinamide-induced model, a multistrain formulation containing seven *Lactobacillus* and *Bifidobacterium* species (5 × 10^10^ CFU), administered alone or in combination with resveratrol, was investigated in Wistar rats. This combination significantly reduced the insulin resistance index; however, a reduction in oxidative stress was observed only in the group co-administered with resveratrol [170]. Other probiotic strategies, including the use of *Lactobacillus* strains such as *L. plantarum*, *L. gasseri*, and *L. fermentum*, have been evaluated in experimental diabetes models, demonstrating antioxidant effects, reduced dysbiosis and normalization of glucose metabolism [171,172]. In murine models of metabolic disorder induced by HFD, the use of probiotic strains such as *Lactobacillus* and *Bifidobacterium* decreased adipose tissue, insulin resistance and inflammation [173,174,175]. In addition, probiotic strains such as *Lactobacillus*, *Streptococcus*, *Clostridium butyricum* and *Bifidobacterium* have demonstrated neuroprotective properties in models of neurological diseases, improving synaptic plasticity and antioxidant capacity, reducing neurodegeneration/neuroinflammation, and preventing anxiety behavior and memory impairment [176,177,178,179,180,181]. CEREBIOME^®^ (*Lactobacillus helveticus* R0052 and *B. longum* R0175) is a specific formulation of a probiotic blend. This treatment significantly improved the anxiety behavior observed in animals with obesity [182]. Another probiotic mixture (*B. bifidum* W23, *B. lactis* W52, *L. acidophilus* W37, *L. brevis* W63, *L. casei* W56, *L. salivarius* W24, and *Lc. lactis* W19, *Lc. lactis* W58) increased indole-3-propionic acid, a neuroprotective agent, and reduced depression in rats fed a HFD [183]. In another study, the prebiotic xylooligosaccharide and probiotic *L. paracasei* improved hippocampal plasticity and brain mitochondrial function and decreased microglial activation, thereby restoring cognitive function in male obese insulin-resistant rats induced by a HFD [184]. The use of *L. plantarum* and inulin also improves gut dysbiosis, oxidative stress and cognitive responses in diabetic rats [185]. This evidence suggests that these strains could also be used to treat neuroinflammation caused by metabolic conditions.

Regarding clinical studies, it has been observed that the use of probiotic strains such as *Lactobacillus*, *Streptococcus*, *Saccharomyces* and *Bifidobacterium* in individuals with T2DM, obesity or metabolic syndrome generally improves insulin resistance, lipid profile, immune system modulation, and metabolic function. Additionally, these interventions have been associated with improved intestinal epithelial barrier integrity, reduced triglyceride levels, and decreased inflammation markers and oxidative stress status [186,187,188,189,190,191]. On the other hand, polyphenols (epigallocatechin gallate, epicatechin gallate, epigallocatechin, gallocatechin, epicatechin and catechin) also exert prebiotic effects by stimulating beneficial bacteria and reducing the incidence of metabolic disorders and cardiometabolic risks [192]. Some polyphenols inhibit harmful bacteria such as *Helicobacter pylori*, *Staphylococcus aureus*, *Escherichia coli* and *Salmonella typhimurium* or hepatitis C virus and *Candida* [193]. In contrast, other polyphenols promote the growth of beneficial bacteria, including *Bifidobacterium* spp., *Lactobacillus* spp., and *Akkermansia muciniphila* [193,194]. Moreover, it has been shown that the consumption of virgin olive oil, which is rich in polyphenols, in obese patients can restore gut microbiota balance and improve insulin sensitivity [195,196]. Whole grains rich in dietary fiber, lignans and phytosterols could also improve insulin sensitivity through modulation of the intestinal microbiota [197]. Furthermore, it has been suggested that polyphenols in combination with prebiotics and exercise may help prevent or attenuate neurogenerative diseases by modulating the gut microbiota and the gut–brain axis, partly due to their ability to cross the BBB [198,199]. Symbiotics, combinations of probiotics and prebiotics with antioxidant-rich components, represent a promising therapeutic strategy due to their potent anti-inflammatory and antioxidant properties. These effects contribute to maintaining a healthy gut environment and protecting against neuroinflammatory pathways [200].

The beneficial effects of probiotics, administered alone or in combination with antioxidants, have been observed in neurological and psychiatric symptoms associated with metabolic conditions in patients. In geriatric subjects with obesity, the consumption of kefir fortified with two strains, *Lactobacillus helveticus* and *Bifidobacterium longum* (dosage 3  ×  10^9^ CFU of each strain), was associated with a reduction in depressive symptoms [201]. In subjects with T2DM and coronary heart disease, co-supplementation with a probiotic (8 × 10^9^ CFU/day of *Lactocare Zisttakhmir Co.*) and vitamin D (50,000 IU every 2 weeks) for 12 weeks significantly decreased depression and anxiety [202]. Similarly, the use of probiotics (8 × 10^9^ CFU/day of *Lactobacillus acidophilus*, *Bifidobacterium bifidum*, *Lactobacillus reuteri* and *Lactobacillus fermentum*; 2  ×  10^9^ CFU/g each) combined with vitamin D (50,000 IU every 2 weeks) for 12 weeks also improved anxiety and depression symptoms in women with polycystic ovary syndrome [203]. The use of the same probiotic protocol combined with selenium co-supplementation (200 μg/day) over the same period also improved depression and anxiety symptoms in these patients [204]. In subjects with metabolic syndrome and chronic schizophrenia, the use of a probiotic (8 × 10^9^ CFU/day containing *Lactobacillus acidophilus*, *Bifidobacterium lactis*, *Bifidobacterium bifidum*, and *Bifidobacterium longum*; 2  ×  10^9^ CFU of each strain; LactoCare^®^, Zisttakhmir Company, Tehran, Iran) combined with selenium (200 μg/day) for 12 weeks improved the severity of neurological symptoms [205]. Additionally, in subjects after a myocardial infarction, the administration of *Lactobacillus rhamnosus* capsules (1.6 × 10^9^ CFU) for 12 weeks significantly reduced depressive symptoms [206]. In pregnant women, the use of a probiotic formulation (Ecologic Barrier) has been used to manage symptoms of prenatal maternal anxiety and depression [207]. In a healthy elderly population, a probiotic formulation containing *L. paracasei*, *L. rhamnosus*, *L. acidophilus*, and *B. lactis* improved cognitive function and reduced inflammation [208]. The use of symbiotics containing *L. acidophilus*, *B. bifidum*, *B. lactis*, and *B. longum*, combined with prebiotics such as fructooligosaccharides, galactooligosaccharides, and inulin, also decreased depressive symptoms in patients undergoing hemodialysis [209]. Regarding clinical trials, a recent randomized, placebo-controlled, parallel-group interventional study is evaluating the effect of a symbiotic supplement (tannins 350 mg and strains including *Lactobacillus acidophilus*, *Lactobacillus casei*, and *Bifidobacterium lactis;* 2 × 10^9^ CFU/g each) on anxiety and depression in subjects with obesity over 3 years [210]. Finally, pharmacological approaches using antioxidant compounds such as resveratrol, curcumin, vitamin C, vitamin E, and β-carotene have been proposed to modulate the gut–brain axis and mitigate the progression of neuroinflammation [211,212].

## 5. Future Research Perspectives

Rather than representing a linear sequence of events, current evidence suggests the existence of a self-amplifying pathological circuit in which metabolic alterations, microbial imbalance, and inflammatory signaling in the brain interact bidirectionally, contributing to disease progression and the limited efficacy of current therapeutic strategies.

In this context, oxidative stress emerges as a central and convergent mechanism linking these processes, representing a promising therapeutic target. Therefore, future research should focus on elucidating the temporal and mechanistic hierarchy of these interactions, as well as on developing antioxidant-based strategies. These approaches include both bioactive compounds and microbiota-derived bacteria with antioxidant capacity, used individually or in combination with prebiotics, with the potential to modulate this pathological axis.

Furthermore, it is essential to strengthen research on how current therapies for metabolic diseases influence the gut–brain axis and neuroinflammation. Progress in this area will enable the development of more effective and accessible interventions, facilitating their application in translational studies.

Finally, it is necessary to develop strategies aimed not only at targeting the initial pathogenic mechanisms but also at limiting the long-term consequences of neuroinflammation and dysbiosis, including their association with neurodegenerative diseases and cancer.

## 6. Conclusions

Defining a precise causal mechanism between metabolic diseases, dysbiosis, and neuroinflammation is complex and challenging. Therefore, in this review, we integrate the main mechanisms that converge on oxidative stress as a common pathophysiological axis.

In this context, antioxidants have emerged as a promising therapeutic strategy, including compounds such as polyphenols, flavonoids, vitamins, and curcumin, as well as bacteria such as *Lactobacillus* and *Bifidobacterium*. The use of antioxidants alone or in combination with strategies targeting microbiota and neuroinflammation represents an innovative and potentially synergistic approach for the treatment of metabolic diseases. However, significant gaps remain in clinical validation, treatment standardization, and the identification of optimal combinations, underscoring the need for future translational studies.

## Figures and Tables

**Table 1:** Bacteria with pro-oxidant potential.

Pro-Oxidant Bacteria	Oxidative Mechanism	Reported Effects
*Enterobacteriaceae* (e.g., *Escherichia coli*, *Enterobacter cloacae*) [132,133].	Their endotoxin (LPS) binds to TLR4 in host cells and activates nicotinamide adenine dinucleotide Phosphate [NADPH] Oxidases (NOX) and inflammatory pathways, generating large amounts of ROS.	They aggravate systemic inflammation and contribute to metabolic dysfunction in obesity.
*Enterococcus faecalis* [131,134,135]	Produces extracellular O_2_^●—^ and H_2_O_2_ to survive, which damages colonic cell DNA and induces the formation of hydroxyl radicals (●HO).	They promote genomic instability and are associated with colorectal cancer.
Sulfate-reducing bacteria (e.g., *Bilophila wadsworthia*, *Desulfovibrio* spp.) [130,136,137].	They use sulfate/sulfite as final electron acceptors and generate H_2_S. In excess, H_2_S damages the mucosal barrier and activates proinflammatory Th1 responses.	They promote colitis and disruption of the intestinal barrier in susceptible hosts.

**Table 2:** Bacteria with antioxidant potential.

Antioxidant Bacteria	Antioxidant Mechanism	Reported Effects
*Lactobacillus* spp. (e.g., *L. casei*, *L. fermentum*, *L. plantarum*) [139,140,141].	They produce SOD and use manganese complexes to neutralize ROS. Prevent ●HO radicals and suppress pro-oxidant intestinal bacteria. Generate GSH, reduce glycation and lipid peroxidation	They reinforce the antioxidant status of the intestinal epithelium and reduce oxidative damage.
*Bifidobacterium* spp. (e.g., *B. longum*, *B. animalis*) [142,143].	They ferment dietary fiber, generating acetate and lactate, which may support barrier integrity and reduce permeability to proinflammatory and oxidative mediators. In obesity, they can decrease LPS, normalizing markers of oxidative stress. They provide B vitamins that act as cofactors in the host’s antioxidant pathways.	They improve mucosal integrity and reduce systemic oxidative inflammation.
*Faecalibacterium prausnitzii* (clostridia group IV) [144,145]	Main producer of butyrate, which nourishes colonocytes and reduces ROS generation by improving barrier function. Secrets microbial anti-inflammatory molecules (MAM), inhibiting NF-κB signaling	Contributes to an anti-inflammatory and antioxidant environment in the colon and improves insulin sensitivity.
*Akkermansia muciniphila* (*Verrucomicrobia*) [146,147]	Degrades mucin and strengthens the mucus layer, limiting the translocation of endotoxins. It is related to lower ROS in the intestinal lumen.	Promotes barrier integrity and reduces oxidative stress in the colon, improving metabolism.

**Table 3:** Antioxidant compounds and supplements targeting gut dysbiosis and neuroinflammation.

Supplement	Effects on Microbiota, Oxidative Stress, and Neuroinflammation	Therapeutic Relevance
Vitamin D (cholecalciferol) [153,154,155].	Restructures the microbiota increase diversity and beneficial genera such as *Akkermansia muciniphila* and *Bifidobacterium*. Reduces intestinal permeability and endotoxemia by strengthening tight junctions. In addition, it activates the vitamin D receptor (VDR) receptors in microglia, modulating the brain’s immune response and decreasing markers of neuroinflammation.	Improves the intestinal barrier, reduces systemic inflammation, and protects cognitive functions by attenuating neuroinflammation.
Vitamins C and E (classic antioxidants) [142,156,157].	Both act as free radical scavengers at the systemic and intestinal levels. Vitamin C, in high doses, enhances SCFA production (e.g., butyrate) via microbiota. Vitamin E (α-tocopherol) promotes SCFA-producing commensals and strengthens the mucosal barrier. Together, they decrease proinflammatory cytokines and reduce neuronal oxidative stress in models of cerebral obesity and neuroinflammation.	They reinforce epithelial integrity, normalize redox metabolism, and preserve neuronal function by mitigating oxidative damage and neuroinflammation.
Polyphenols (e.g., resveratrol, grape polyphenols) [158,159,160].	They directly neutralize ROS and regulate NF-κB, modulating inflammatory circuits. They function as prebiotics, increasing beneficial genera (*Lactobacillus*, *Bifidobacterium*, *Akkermansia*), reducing *Enterobacteriaceae*, and reducing barrier permeability. In models of metabolic syndrome, they limit microglia activation and decrease markers of neuroinflammation.	They restore the microbial ecosystem, improve insulin sensitivity, and protect cognitive functions by reducing oxidative stress and neuroinflammation.
Omega-3 fatty acids (Eicosapentaenoic Acid/Docosahexaenoic Acid (EPA/DHA)) [161,162,163].	They inhibit proinflammatory pathways (e.g., NF-κB) and generate resolvins; they promote butyrate-producing bacteria and reduce pro-inflammatory pathogens. They strengthen tight junctions in the intestine and promote regulatory T cells. Metabolites cross the BBB to attenuate pro-inflammatory cytokines and decrease microglial activation.	They maintain intestinal homeostasis, reduce systemic inflammation, and preserve neurocognitive function by modulating the gut–brain axis.
Curcumin (turmeric polyphenol) [25,164,165,166].	It is biotransformed in the colon and enhances beneficial genera (*Lactobacillus*, *Bifidobacterium*), suppressing *Enterobacteriaceae* and *Prevotellaceae*. It reduces permeability and circulating LPS. It crosses the BBB in low proportions, inhibits microglia and astroglia activation, and decreases markers of cerebral oxidative stress in models of neurodegeneration and obesity.	It optimizes barrier function, normalizes the inflammatory profile, and preserves memory and other cognitive functions by mitigating neuroinflammation and oxidative stress.

## Data Availability

No new data were created or analyzed in this study. Data sharing is not applicable to this article.

## Abbreviations

The following abbreviations are used in this manuscript:

APN	Adiponectin
AGEs	Advanced Glycation End Products
ATP	Adenosine Triphosphate
BAT	Adenosine Triphosphate
BBB	Blood–Brain Barrier
BDNF	Brain-Derived Neurotrophic Factor
CFU	Colony-Forming Units
CAT	Catalase
EPA/DHA	Eicosapentaenoic Acid/Docosahexaenoic Acid
FMT	Fecal Microbiota Transplantation
GLP-1	Glucagon-Like Peptide-1
GPR41	G-Protein-Coupled Receptor 41
GPR43	G-Protein-Coupled Receptor 43
GPCR	G-Protein-Coupled Receptor
GPx	Glutathione Peroxidase
GSH	Reduced Glutathione
H_2_O_2_	Hydrogen Peroxide
H_2_S	Hydrogen Sulfide
HFD	High-Fat Diet
HDAC3	Histone Deacetylase 3
HDL	High-Density Lipoprotein
IL-1β	Interleukin-1 Beta
IL-6	Interleukin-6
IL-18	Interleukin-18
IRAK1/2	Interleukin-1 Receptor-Associated Kinase 1/2
JNK	c-Jun N-terminal Kinase
Keap1	Kelch-like ECH-Associated Protein 1
LPS	Lipopolysaccharide(s)
LDL	Low-Density Lipoprotein
MCP-1	Monocyte Chemoattractant Protein-1
MD-2	Myeloid Differentiation Factor 2
MGB	Microbiota–Gut–Brain Axis
MPO	Myeloperoxidase
MS	Metabolic Syndrome
MyD88	Myeloid Differentiation Primary Response 88
NF-κB	Nuclear Factor Kappa-Light-Chain-Enhancer of Activated B Cells
NLRP3	NOD-Like Receptor Family, Pyrin Domain Containing 3
NOX4	NADPH Oxidase 4
NPY	Neuropeptide Y
Nrf2	Nuclear Factor Erythroid 2-Related Factor 2
oxLDL	Oxidized Low-Density Lipoprotein
POMC	Proopiomelanocortin
PKC	Protein Kinase C
ROS	Reactive Oxygen Species
SCFAs	Short-Chain Fatty Acids
SOD	Superoxide Dismutase
STZ	Streptozotocin
TLR4	Toll-Like Receptor 4
TNF-α	Tumor Necrosis Factor Alpha
TIRAP	Toll/Interleukin-1 Receptor Domain-Containing Adaptor Protein
TRAF6	TNF Receptor-Associated Factor 6
VDR	Vitamin D Receptor
WAT	White Adipose Tissue
